# Effects of phentermine / topiramate extended-release, phentermine, and placebo on ambulatory blood pressure monitoring in adults with overweight or obesity: A randomized, multicenter, double-blind study

**DOI:** 10.1016/j.obpill.2024.100099

**Published:** 2024-01-08

**Authors:** Harold E. Bays, Daniel S. Hsia, Lan T. Nguyen, Craig A. Peterson, Santosh T. Varghese

**Affiliations:** a Louisville Metabolic and Atherosclerosis Research Center, University of Louisville School of Medicine, 3288 Illinois Avenue, Louisville, KY, 40213, USA; b Pennington Biomedical Research Center, Baton Rouge, LA, USA; c VIVUS LLC, Campbell, CA, USA

**Keywords:** Ambulatory blood pressure monitoring, Hypertension, Obesity, Phentermine, Topiramate

## Abstract

**Background:**

A fixed-dose combination of phentermine and extended-release topiramate (PHEN/TPM - approved for weight management) has demonstrated in-clinic reduction of blood pressure (BP). Ambulatory BP monitoring (ABPM) may be a better predictor of cardiovascular disease risk than in-clinic BP.

**Methods:**

This randomized, multicenter, double-blind study enrolled 565 adults with overweight/obesity. Inclusion criteria included participants willing to wear ABPM device for 24 h. Exclusion criteria included screening blood pressure >140/90 mmHg and antihypertensive medications not stable for 3 months prior to randomization. Participants received placebo (n = 184), phentermine 30 mg; (n = 191), or PHEN 15 mg/TPM 92 mg; (n = 190). 24-hour ABPM was performed at baseline and at week 8. The primary endpoint was mean 24-h systolic BP (SBP) as measured by ABPM, in the per protocol population.

**Results:**

Participants were mostly female (73.5 ​%) and White (81.6 ​%), with a mean age of 53.4 years; 32.4 ​% had no hypertension diagnosis or treatment, 62.5 ​% had hypertension using 0 to 2 antihypertensive medications, and 5.1 ​% had hypertension using ≥ 3 antihypertensive medications. Baseline mean SBP/diastolic BP (DBP) was 123.9/77.6 ​mmHg. At week 8, mean SBP change was −0.1 ​mmHg (placebo), +1.4 ​mmHg (phentermine 30 ​mg), and −3.3 ​mmHg (PHEN/TPM). Between-group difference for PHEN/TPM versus placebo was −3.2 ​mmHg (95 ​% CI: -5.48, -0.93 ​mmHg; p ​= ​0.0059). The between-group difference for PHEN/TPM versus phentermine 30 ​mg was −4.7 ​mmHg (95 ​% CI: −6.96, −2.45 ​mmHg; p ​< ​0.0001). Common (>2 ​% in any treatment group) adverse events (i.e., dry mouth, constipation, nausea, dizziness, paresthesia, dysgeusia, headache, COVID-19, urinary tract infection, insomnia, and anxiety) were mostly mild or moderate.

**Conclusions:**

In this randomized, multicenter, double-blind ABPM study, PHEN/ TPM reduced SBP compared to either placebo or phentermine 30 mg (Funding: Vivus LLC; ClinicalTrials.gov: NCT05215418).

## Graphical abstract

**Figure:**
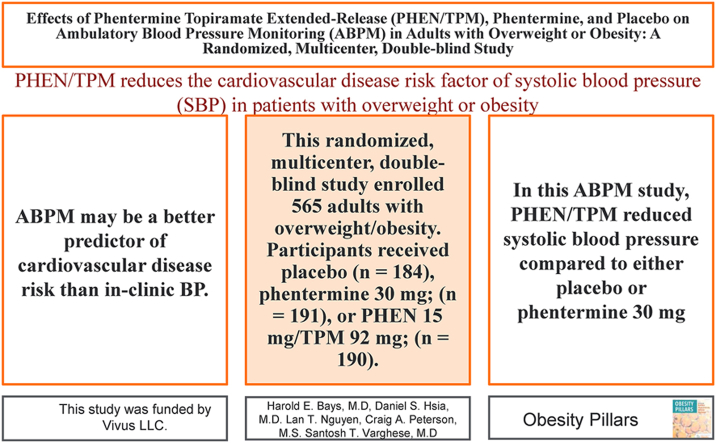

## 1 Introduction

One of the more common adiposopathic complications of obesity is an increase in blood pressure [1]. Patients with obesity and hypertension are at increased risk for adverse clinical consequences such as cardiovascular and renal disease [2], prompting the recommendation that in addition to antihypertensive medications, patients with obesity may benefit from healthful nutrition, increased physical activity, anti-obesity medications, and possibly bariatric surgery [3]. Use of anti-obesity medications among patients with obesity and hypertension can sometimes present challenges. While anti-obesity medications such as orlistat and glucagon-like peptide (GLP) receptor agonists may reduce blood pressure, the prescribing information for sympathomimetic agents warn of potential adverse reactions in raising blood pressure [1].

The fixed dose combination of immediate release phentermine and extended-release topiramate (PHEN/TPM) was approved in the US in 2012 as an adjunct to a reduced calorie diet and increased physical activity for chronic weight management regarding (a) adults with an initial body mass index (BMI) of ≥ 30 kg/m^2^ (obese) or ≥ 27 kg/m^2^ (overweight) in the presence of at least 1 weight-related comorbidity such as hypertension, type 2 diabetes mellitus (T2DM), or dyslipidemia or (b) pediatric patients aged 12 years and older with BMI in the ≥ 95th percentile standardized for age and sex (Qsymia prescribing information. Campbell, CA: VIVUS LLC; 2023 https://qsymia.com/patient/include/media/pdf/prescribing-information.pdf?v=0422).

One of the components of PHEN/TPM is topiramate. Topiramate is a sulfamate-substituted monosaccharide derivative of D-fructose approved for the treatment of epilepsy and migraine prevention, which was also evaluated for treatment of type 2 diabetes mellitus and obesity. However, the degree of glucose improvement of topiramate alone was not sufficient to achieve an indicated use as an anti-diabetes agent and the degree of weight reduction with topiramate alone was not sufficient to achieve an indicated use as an anti-obesity medication [4]. In a randomized, placebo-controlled trial in participants with obesity, topiramate reduced body weight and blood pressure. Adverse events included paresthesia, fatigue, taste perversion, loss of appetite, and difficulty with concentration and attention [5]. Mechanistically, topiramate inhibits sodium and calcium voltage-gated channels, inhibits glutamate receptors, inhibits carbonic anhydrase, and enhances gamma-aminobutyric acid (GABA) receptors [6] Inhibition of renal carbonic anhydrase is associated with diuresis, potentially helping to account for a reduction in blood pressure with topiramate [7].

The other component of PHEN/TPM is phentermine, which is a sympathomimetic agent. Phentermine as monotherapy is typically used at a higher dose (30 mg) than is found in PHEN/TPM, and is contraindicated in patients with cardiovascular disease or uncontrolled hypertension [8]. Moreover, the effects of higher dose phentermine monotherapy on blood pressure have not been reported in controlled trials, potentially raising concerns about a potential increase in blood pressure with higher dose phentermine monotherapy. That said, PHEN/TPM is unique in that the doses of the phentermine component are less than when phentermine is used as monotherapy, and the combination also contains topiramate, which inhibits carbonic anhydrase enzymes and may lower blood pressure [9]. Clinical trial experience suggests that treatment with PHEN/TPM may not only reduce body weight, but also reduce in-clinic blood pressure compared to placebo. PHEN/TPM may also mildly increase heart rate (1–2 bpm) [10], which is a finding also reported with other anti-obesity medications such as GLP -1 receptor agonists [1].

Prior to this study, the BP data collected to date for PHEN/TPM was obtained only via in-clinic visits, typically in the morning. However, ambulatory BP monitoring (ABPM) (i.e., performed out of the office setting via BP readings every 15–30 min intervals for 24–48 h) is considered superior to a single office BP measurement for an overall assessment of BP [9]. Because ABPM may better predict target organ damage and cardiovascular disease risk, some believe ABPM is the gold standard BP measurement technique – especially for patients with variable BP readings or patients with suspected “white coat” or “masked” hypertension [9]. This randomized, multicenter, double-blind study evaluated the effects of PHEN/TPM on blood pressure as measured by ABPM over 24 h.

## 2 Methods

### 2.1 Design

This was a randomized, multi-center, double-blind, placebo- and active-controlled study that evaluated the effect of PHEN/TPM (PHEN (phentermine) 15 mg/TPM (topiramate) 92 mg), phentermine 30 mg, and placebo (1:1:1 randomization ratio) on blood pressure readings via 24-h ABPM. Phentermine was administered as a hydrochloride (HCl) formulation. The PHEN/TPM was administered as a combination of immediate release phentermine HCl and topiramate extended release. Study treatments were administered orally once daily (morning) for 8 weeks. For participants in the PHEN/TPM group, dosages were titrated up to the assigned levels at weekly intervals during weeks 1–3. Participants assigned to the PHEN/TPM group were provided blinded blister cards that contained the protocol-directed escalation of study drug doses. At each study site, participants also received protocol-directed lifestyle counseling, which included advice on a reduced calorie diet and increase in physical activity.

Twenty-four-hour ABPM recordings were performed at baseline/randomization and again at week 8 or early termination (ET). Blood pressure and heart rate were measured every 20 min from 06:00 to 22:00 h, and every 30 min from 22:01 to 05:59 h for 24 consecutive hours, while participants continued normal routine activities. The 24-h ABPM data was read and analyzed in a blinded manner by a central reader, using validated software. Concomitant medications for management of blood pressure or other weight-related comorbidities were fixed in number, dose, and frequency for the duration of the study unless specific “rescue” criteria were met for BP (≥160/100 mmHg, or ≤ 100/60 mmHg and symptoms of hypotension). Safety was evaluated by analysis of adverse events, changes in laboratory parameters, changes in electrocardiogram (ECG), physical exam findings, vital signs, and requirement for rescue therapy for blood pressure and T2DM.

### 2.2 Participants

Inclusion criteria included adults with a BMI ≥ 27 kg/m^2^ and a medical diagnosis of at least 1 weight-related comorbidity (i.e., hypertension, dyslipidemia, T2DM, prediabetes, or obstructive sleep apnea), who were ambulatory and willing and able to wear an ABPM device for 24 h. Participants were excluded if they had screening blood pressure > 140/90 mmHg, use of antihypertensive medication not stable for at least 3 months prior to randomization, a need to perform strenuous manual labor during ABPM recording, night shift work hours, or any clinically significant renal, pulmonary, hepatic, psychiatric or other condition that in the opinion of the investigator would contraindicate the administration of study drug, affect compliance, interfere with study evaluations, or confound the interpretation of study results. The full list of inclusion and exclusion criteria is provided in the supplementary appendix. Study subjects were recruited according to local practices of the participating research sites (e.g., database, advertisements).

### 2.3 Study oversight

This study was conducted from 25 January 2022 to 17 April 2023, at 29 sites in the United States. The study was initiated after approval of the Institutional Review Board, after proper consent from the participants, and conducted in compliance with the principles of the Declaration of Helsinki, International Conference on Harmonisation (ICH), Good Clinical Practices (GCP) guidelines, and applicable US Code of Federal Regulations. The sponsor designed and wrote the protocol, provided medical monitoring and overall study oversight. Various contract research organizations supported other aspects of the study.

### 2.4 Endpoints and statistical methods

The primary study endpoint of change from baseline to week 8 in mean SBP from 24 h ABPM was evaluated in the per protocol (PP) population which included randomized participants who received at least 1 dose of study drug; were compliant to study treatment; had last dose of study drug administered on the same date that final ABPM was initiated; had baseline and final ABPM with at least 23.5 h of data; had at least 75 % of 24-h ABPM readings (or, equivalently, 48 readings during the 24-h period) that were not missing; and did not have major protocol deviations that had impact on ABPM readings. The reason the per protocol analysis was utilized instead of an intent-to-treat analysis was due to regulatory requirement from the Food and Drug Administration, given this study primarily evaluated blood pressure as a safety endpoint, and not a weight reduction efficacy endpoint.

Analyses were conducted utilizing Statistical Analysis Software (SAS) Version 9.4. An analysis of covariance (ANCOVA) model was used to evaluate between-group differences (PHEN/TPM vs. placebo and PHEN/TPM vs. phentermine 30 ​mg) in changes from baseline in mean 24-h SBP. Factors included in the model were study treatment, sex, age, and hypertensive status. Baseline values of 24-h mean SBP were adjusted in the model as a covariate. For comparisons between PHEN/TPM and comparator groups, an upper 95 ​% confidence bound of +3 ​mmHg was the study-specified non-inferiority margin, and an upper 95 ​% confidence bound of 0 ​mmHg was the study-specified superiority margin. Secondary endpoints evaluated changes from baseline to week 8 in SBP measured in-clinic, mean 24-h ABPM DBP, and DBP measured in-clinic in the PP population using a similar ANCOVA model structure to that of the primary analysis.

Exploratory endpoints evaluated changes from baseline to week 8 in hourly BP, daytime and nighttime BP, nighttime BP dipping, and heart rate. These were performed on the PP population using the observed case data and ANCOVA models in a similar manner to the primary and secondary endpoints.

This study was designed to maintain an overall study-wise type I error rate of α = 0.05. A hierarchical gatekeeping approach was to be used to control the family-wise type I error of the primary and secondary endpoints.

Safety analyses were performed on the safety population (all randomized participants who received at least 1 dose of study drug). The safety variables included adverse events (AEs), changes in laboratory results, vital signs, physical examinations, and ECGs, and requirement for rescue therapy for BP and T2DM.

## 3 Results

### 3.1 Study population

**Fig. 1:**
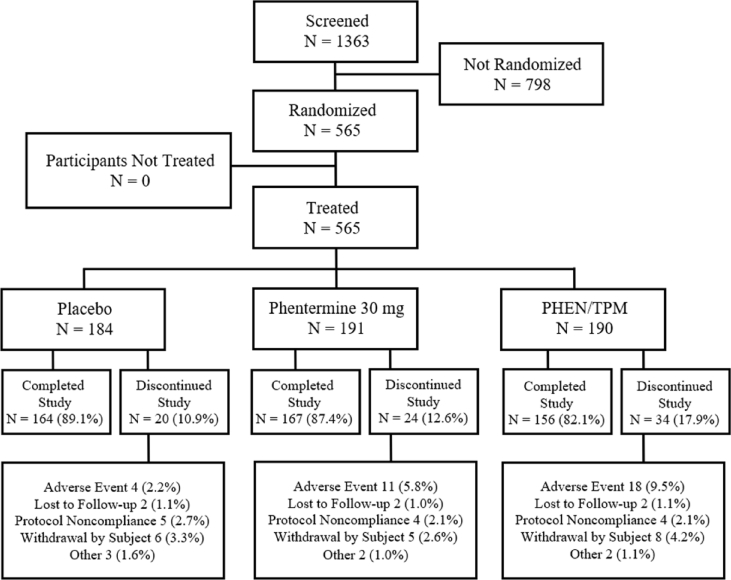
Disposition of Participants for Protocol OB-409, All Screened Participant Population The percentages are based on the number of randomized participants under each treatment group.

A total of 1363 participants were screened and 565 were randomized to receive either placebo (n = 184), phentermine 30 mg (n = 191), or PHEN/TPM 15 mg/92 mg (n = 190). The majority of the participants in each group completed all study visits (89.1 %, 87.4 %, and 82.1 % in the placebo, phentermine, and PHEN/TPM groups, respectively). The main reasons for discontinuation from the study overall included AEs (2.2 %, 5.8 %, and 9.5 % in the placebo, phentermine, and PHEN/PM groups, respectively), withdrawal by participant (3.3 %, 2.6 %, and 4.2 % in the placebo, phentermine, and PHEN/TPM groups, respectively), and protocol noncompliance (2.7 %, 2.1 %, and 2.1 % in the placebo, phentermine, and PHEN/TPM groups, respectively) as indicated in Fig. 1.

Participants had a mean (SD) age of 53.4 (12.29) years, 73.5 % were female, 81.6 % were White, 14.3 % were Black, and 26.4 % were Hispanic/Latino. At baseline 32 % of participants had no prior history of hypertension, 62.5 % had hypertension treated with 0–2 antihypertensive medications, and 5.1 % had hypertension treated with ≥ 3 antihypertensive medications. Baseline mean SBP/DBP was 123.9/77.6 mmHg; mean weight and BMI were 99.6 kg and 35.8 kg/m^2^, respectively. Type 2 diabetes was present in 11.3 % of participants and prediabetes was present in 13.5 % of participants. Table 1.

**Table 1:** Summary of demographics and baseline characteristics- ITT population.

Characteristics	Placebo (n = 184)	Phentermine 30 mg (n = 191)	PHEN/TPM 15 mg/92 mg (n = 190)	Overall (N = 565)
**Age (Years),**				
Mean (SD)	53.3 (11.36)	53.3 (12.12)	53.6 (13.34)	53.4 (12.29)
**Sex, n (%)**				
Male	47 (25.5)	49 (25.7)	54 (28.4)	150 (26.5)
Female	137 (74.5)	142 (74.3)	136 (71.6)	415 (73.5)
**Race, n (%)**				
White	140 (76.1)	167 (87.4)	154 (81.1)	461 (81.6)
Black or African American	39 (21.2)	17 (8.9)	25 (13.2)	81 (14.3)
American Indian or Alaska Native	2 (1.1)	1 (0.5)	2 (1.1)	5 (0.9)
Asian	0	3 (1.6)	2 (1.1)	5 (0.9)
Native Hawaiian or Other Pacific Islander	1 (0.5)	1 (0.5)	1 (0.5)	3 (0.5)
Multiple	2 (1.1)	0	2 (1.1)	4 (0.7)
Not Reported	0	2 (1.0)	4 (2.1)	6 (1.1)
**Ethnicity, n (%)**				
Hispanic or Latino	47 (25.5)	50 (26.2)	52 (27.4)	149 (26.4)
Not Hispanic or Latino	137 (74.5)	141 (73.8)	138 (72.6)	416 (73.6)
Unknown	0	0	0	0
**Hypertensive Status, n (%)**				
No Medical Diagnosis of or Treatment for Hypertension	58 (31.5)	61 (31.9)	64 (33.7)	183 (32.4)
Medical Diagnosis of Hypertension Treated with 0–2 Antihypertensive Medications	117 (63.6)	120 (62.8)	116 (61.1)	353 (62.5)
Medical Diagnosis of Hypertension Treated with 3 or More Antihypertensive Medications	9 (4.9)	10 (5.2)	10 (5.3)	29 (5.1)
**Diabetes Status, n (%)**				
Type 2 diabetes	20 (10.9)	19 (9.9)	25 (13.2)	64 (11.3)
Prediabetes	25 (13.6)	24 (12.6)	27 (14.2)	76 (13.5)
**SBP (mmHg) as Measured in Clinic**				
Mean (SD)	124.5 (11.23)	123.4 (10.38)	123.9 (9.82)	123.9 (10.48)
**DBP (mmHg) as Measured in Clinic**				
Mean (SD)	78.3 (7.71)	77.0 (7.98)	77.5 (7.63)	77.6 (7.78)
**Weight (kg)**				
Mean (SD)	100.4 (21.52)	99.5 (21.58)	98.8 (20.84)	99.6 (21.29)
**Height (cm)**				
Mean (SD)	166.1 (10.08)	166.6 (9.51)	165.7 (9.16)	166.2 (9.58)
**Body Mass Index (kg/m**^**2**^**)**				
Mean (SD)	36.2 (6.17)	35.4 (5.88)	35.7 (6.20)	35.8 (6.08)

Abbreviations: DBP = diastolic blood pressure; Max = maximum; Min = minimum; SBP = systolic blood pressure; SD = standard deviation.

Baseline is defined as the last non-missing observation obtained prior to dispensing of the first study drug.

The percentage is based on the number of participants under each treatment group in the ITT Population.

### 3.2 Primary efficacy, secondary, and exploratory outcomes

**Fig. 2:**
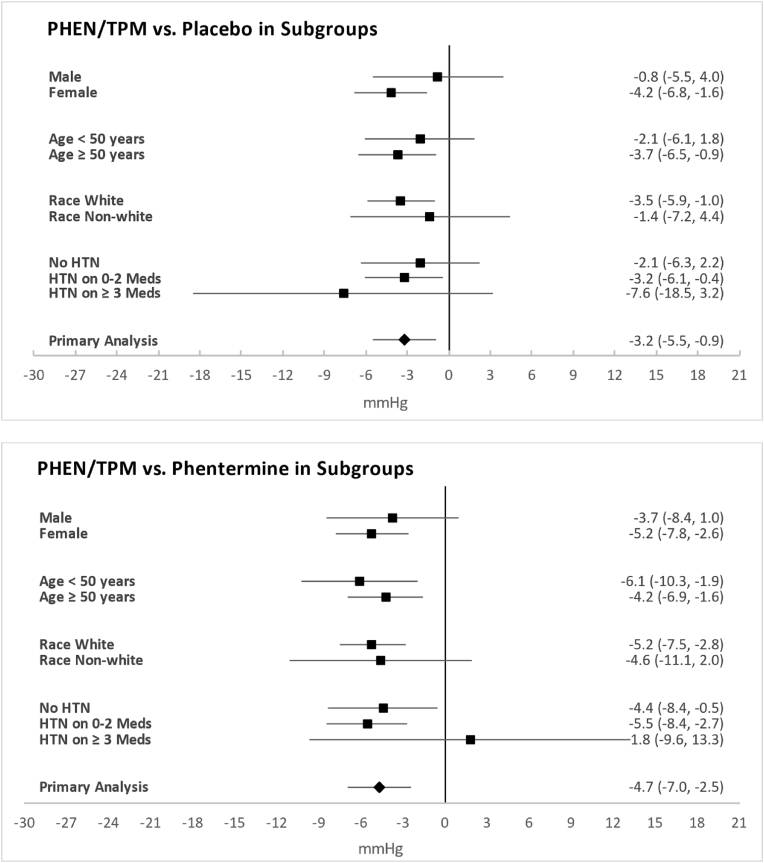
Forest Plots of ANCOVA Analysis of Change from Baseline to Week 8/ET in Mean SBP as measured by 24-h ABPM – PHEN/TPM versus Placebo and PHEN/TPM versus Phentermine – Subgroup Analysis Abbreviations: ABPM = ambulatory blood pressure monitoring; ANCOVA = analysis of covariance; ET = early termination; HTN = hypertension; Meds = medications; SBP = systolic blood pressure.

ANCOVA analysis of the primary study endpoint of mean 24-h change from baseline in SBP at week 8 demonstrated least squares (LS) mean changes of −0.1 ​mmHg for placebo, +1.4 ​mmHg for phentermine, and −3.3 ​mmHg for PHEN/TPM. The between-group difference for PHEN/TPM versus placebo was −3.2 ​mmHg (95 ​% CI: -5.48, -0.93 ​mmHg; p ​= ​0.0059), which met the margins for both non-inferiority (upper 95 ​% confidence bound of < +3 ​mmHg) and superiority (upper 95 ​% confidence bound of < 0 ​mmHg). The between-group difference for PHEN/TPM versus phentermine was −4.7 ​mmHg (95 ​% CI: −6.96, −2.45 ​mmHg; p ​< ​0.0001) also met the margins for both non-inferiority and superiority. The between-group difference for phentermine versus placebo was 1.5 ​mmHg (95 ​% CI: −0.73, 3.73 ​mmHg; p ​= ​0.1867) did not meet the margins for non-inferiority (Table 2). Fig. 2 supports generally similar directionality of effects of PHEN/TPM irrespective of sex, age, race, and pre-study hypertension status.

**Table 2:** Least-squares (LS) mean change from baseline treatment difference from placebo and phentermine in mean blood pressure for primary and secondary endpoints.

	Placebo (n = 130)	Phentermine 30 mg (n = 133)	PHEN/TPM 15 mg/92 mg (n = 122)	PHEN/TPM-Placebo: LS Mean (95 % CI)	PHEN/TPM-Phentermine: LS Mean (95 % CI)
**Primary Endpoint**					
**Mean SBP by 24-hr ABPM, mmHg**					
Baseline Mean (SD)	120.8 (11.23)	119.4 (12.21)	118.7 (12.04)	−3.2 (−5.48, −0.93)	−4.7 (−6.96, −2.45)
LS Mean Change (SE)	−0.1 (1.04)	1.4 (1.05)	−3.3 (1.07)		
**Secondary Endpoints**					
**SBP Measured in Clinic, mmHg**					
Baseline Mean (SD)	124.3 (11.22)	122.5 (10.62)	123.9 (10.22)	−2.6 (−5.09, −0.20)	−3.2 (−5.58, −0.73)
LS Mean Change (SE)	−1.7 (1.12)	−1.2 (1.13)	−4.3 (1.15)		
**Mean DBP by 24-hr ABPM, mmHg**					
Baseline mean (SD)	73.3 (6.84)	73.1 (7.82)	71.6 (8.98)	1.2 (−0.17, 2.58)	−1.5 (−2.89, −0.17)
LS Mean Change (SE)	−0.4 (0.63)	2.4 (0.64)	0.8 (0.64)		
**DBP Measured in Clinic, mmHg**					
Baseline mean (SD)	78.4 (7.88)	77.3 (8.05)	77.8 (7.92)	−0.2 (−1.95, 1.53)	−2.3 (−4.06, −0.61)
LS Mean Change (SE)	−1.1 (0.80)	1.0 (0.81)	−1.3 (0.82)		

DBP = diastolic blood pressure; LS = least squares; SBP = systolic blood pressure SD = standard deviation; SE = standard error.

Comparisons between PHEN/TPM and placebo met the non-inferiority threshold for all 3 secondary endpoints (mean 24-h ABPM DBP, in-clinic SBP, and in-clinic DBP), but only met the superiority threshold for in-clinic SBP. Comparisons between PHEN/TPM and phentermine also met both the superiority and non-inferiority thresholds for each of the secondary endpoints; however, these results may be considered exploratory since comparisons between PHEN/TPM and placebo did not demonstrate superiority for secondary endpoints based on DBP (Table 2).

For exploratory endpoints, comparisons between PHEN/TPM and placebo for variables based on SBP, all but mean nighttime SBP had comparison-wise CIs that met the superiority criteria. By contrast, comparisons based on DBP did not meet superiority criteria for any endpoints and did not achieve non-inferiority for nighttime DBP (Table 3). Comparison-wise treatment differences between PHEN/TPM and phentermine met non-inferiority criteria for all BP comparisons and met superiority criteria for all but mean nighttime DBP as measured by ABPM (Table 3).

**Table 3:** Least-squares (LS) mean change from baseline treatment difference from placebo and phentermine in exploratory endpoints.

Exploratory Endpoints	Placebo (n = 130)	Phentermine 30 mg (n = 133)	PHEN/TPM 15 mg/92 mg (n = 122)	PHEN/TPM-Placebo: LS Mean (95 % CI)	PHEN/TPM-Phentermine: LS Mean (95 % CI)
**Mean Daytime SBP by 24-hr ABPM, mmHg**					
Baseline Mean (SD)	125.1 (11.49)	123.1 (12.46)	122.1 (12.02)	−3.9 (−6.27, −1.50)	−4.9 (−7.27, −2.57)
LS Mean Change (SE)	−0.5 (1.08)	0.5 (1.10)	−4.4 (1.12)		
**Mean Nighttime SBP 24-hr ABPM, mmHg**					
Baseline Mean (SD)	108.0 (13.68)	108.0 (14.87)	108.3 (15.06)	−0.5 (−3.33, 2.35)	−4.3 (−7.10, −1.47)
LS Mean Change (SE)	1.0 (1.30)	4.8 (1.31)	0.5 (1.34)		
**Mean Daytime DBP by 24-hr ABPM, mmHg**					
Baseline mean (SD)	76.4 (7.04)	76.1 (8.24)	74.3 (9.18)	0.9 (−0.56, 2.41)	−1.6 (−3.11, −0.17)
LS Mean Change (SE)	−0.2 (0.67)	2.4 (0.68)	0.8 (0.69)		
**Mean Nighttime DBP by 24-hr ABPM, mmHg**					
Baseline mean (SD)	63.8 (9.24)	64.3 (8.83)	63.5 (10.53)	1.7 (−0.22, 3.60)	−1.5 (−3.40, 0.39)
LS Mean Change (SE)	0.0 (0.87)	3.2 (0.88)	1.7 (0.90)		
**Mean Nighttime “Dipping” of SBP by 24-hr ABPM, mmHg**					
Baseline mean (SD)	13.6 (8.41)	12.2 (8.47)	11.3 (8.69)	−3.1 (−4.86, −1.32)	−0.3 (−2.08, 1.41)
LS Mean Change (SE)	−0.9 (0.80)	−3.6 (0.81)	−4.0 (0.83)		
**Mean Nighttime “Dipping” of DBP by 24-hr ABPM, mmHg**					
Baseline mean (SD)	16.6 (9.78)	15.4 (8.88)	14.5 (9.67)	−1.8 (−3.89, 0.35)	−0.3 (−2.40, 1.80)
LS Mean Change (SE)	0.0 (0.97)	−1.5 (0.98)	−1.8 (1.00)		
**Mean Heart Rate by 24-hr ABPM, beats/min**					
Baseline mean (SD)	78.1 (9.0)	76.2 (8.53)	77.2 (9.10)	3.6 (2.08, 5.15)	−3.6 (−5.15, −2.10)
LS Mean Change (SE)	−1.0 (0.70)	6.2 (0.71)	2.6 (0.72)		
**Mean Percent Change Body Weight, %**					
Baseline mean (SD)	100.4 (21.5)	99.5 (21.6)	98.8 (20.8)	−3.9 (−4.85, −2.97)	−0.1 (−1.07, 0.79)
LS Mean Change (SE)	0.0 (0.43)	−3.8 (0.44)	−3.9 (0.44)		

DBP = diastolic blood pressure; LS = least squares; SBP = systolic blood pressure SD = standard deviation; SE = standard error.

**Fig. 3:**
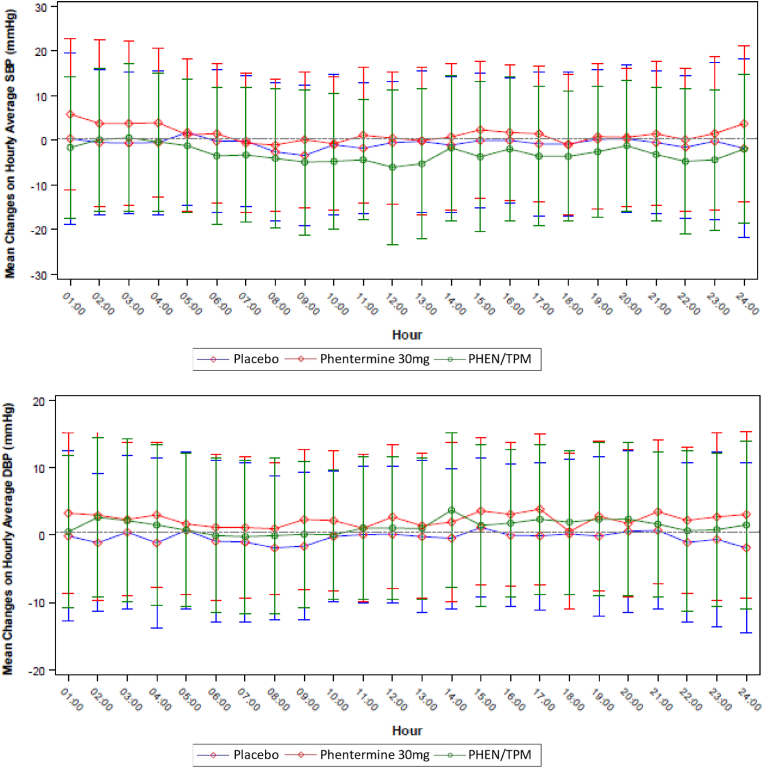
Mean (SD) Change from Baseline to Week 8 in Hourly Average SBP and DBP DBP = diastolic blood pressure; SBP = systolic blood pressure.

Compared to placebo, the PHEN/TPM group had reduced nighttime dipping of SBP [‘dipping’ change of SBP was −3.1 mmHg (95 % CI: −4.86, −1.32 mmHg)]. For PHEN/TPM versus phentermine, the between-group difference was −0.3 mmHg (95 % CI: −2.08, 1.41 mmHg). These observations are consistent with the larger reductions of SBP in the PHEN/TPM group (vs. placebo) in the daytime than at nighttime. Participants in the phentermine group also demonstrated reduced nighttime SBP dipping compared to placebo. The between-group difference was −2.8 mmHg (95 % CI: −4.48, −1.03 mmHg). For this comparison, however, reduced nighttime dipping appears to be the result of greater increases in nighttime SBP than daytime SBP with phentermine. There were no significant differences between treatment groups for nighttime dipping of DBP (Table 3). Mean hourly changes in SBP and DBP from baseline to week 8/ET are summarized in Fig. 3.

Both PHEN/TPM and phentermine 30 mg resulted in increases in mean heart rate as measured by ABPM by 2.6 and 6.2 bpm, respectively; whereas, placebo resulted in a 1.0 bpm reduction in heart rate. Reductions in body weight were comparable in the PHEN/TPM and phentermine groups (3.9 % and 3.8 %, respectively) vs. 0.0 % in the placebo group (Table 3). The observed body weight changes regarding all of the interventions should consider that this study was not intended to evaluate weight reduction in that it lasted only 8 weeks, while most previously published studies of PHEN/TPM primarily evaluated body weight changes 28 weeks or longer [11].

### 3.3 Safety

The incidence of participants reporting at least 1 treatment-emergent adverse event (TEAE) was 17.9 %, 40.3 %, and 41.1 % in the placebo, phentermine, and PHEN/TPM groups, respectively. TEAEs considered by investigator to be drug-related were reported by 9 (4.9 %), 57 (29.8 %), and 55 (28.9 %) participants in the placebo, phentermine, and PHEN/TPM groups, respectively (Table 4). The most common (>2 % in any treatment group) TEAEs included dry mouth, constipation, nausea, dizziness, paresthesia, dysgeusia, headache, COVID-19, urinary tract infection, insomnia, and anxiety. Most of these events were mild or moderate across treatment groups. Severe TEAEs were reported in 1 participant (0.5 %) in the placebo group (white blood cell count decreased), 3 participants (1.6 %) in the phentermine group (1 participant with insomnia; 1 participant with nausea, dizziness, headache, and hypertension; and 1 participant with angina pectoris), and 2 participants (1.1 %) in the PHEN/TPM group (1 participant with dry mouth and 1 participant with paresthesia). All severe TEAEs were considered as study drug related except for white blood cell count decrease. The events of nausea, dizziness, headache, and hypertension were still ongoing at the time the participant completed the study. The other events resolved. The number (%) of participants who withdrew from the study because of TEAEs was 4 (2.2 %) in the placebo group, 11 (5.8 %) in the phentermine group, and 18 (9.5 %) in the PHEN/TPM group. One SAE (angina pectoris) was reported in a participant in the phentermine group. This SAE was considered by the investigator to be not related to study drug and the participant completed the study. No participants died during the study. There were few clinically important findings in laboratory changes, vital signs, physical examination findings, or ECG results. A higher percentage of participants in the PHEN/TPM group (33.5 %) had shifts in serum carbon dioxide (bicarbonate) from normal at baseline to low at week 8/ET compared to the phentermine group (8.4 %) and placebo group (5.8 %). The percentage of participants who had shifts in serum creatinine from normal at baseline to high at week 8/ET were 7.6 % the PHEN/TPM group, 4.5 % in the phentermine group, and 0.6 % in the placebo group. Observed shifts in carbon dioxide and creatinine are consistent with previous data on PHEN/TPM.

**Table 4:** Overall summary of treatment-emergent adverse events and summary TEAE with incidence of ≥ 2 % of participants in any treatment group, safety population.

Category, n (%)	Placebo (n = 184)	Phentermine 30 mg (n = 191)	PHEN/TPM 15 mg/92 mg (n = 190)	Overall (N = 565)
Participants with any TEAE	33 (17.9)	77 (40.3)	78 (41.1)	188 (33.3)
Participants with any TEAE related to study drug	9 (4.9)	57 (29.8)	55 (28.9)	121 (21.4)
Participants with serious TEAE, by preferred term	0	1 (0.5)	0	1 (0.2)
Angina Pectoris	0	1 (0.5)	0	1 (0.2)
Participants with serious TEAE related to study drug	0	0	0	0
Participants with severe TEAE	1 (0.5)	3 (1.6)	2 (1.1)	6 (1.1)
TEAE > 2 % in any treatment group				
Dry mouth	1 (0.5)	22 (11.5)	22 (11.6)	45 (8.0)
Constipation	2 (1.1)	11 (5.8)	7 (3.7)	20 (3.5)
Nausea	2 (1.1)	5 (2.6)	5 (2.6)	12 (2.1)
Dizziness	1 (0.5)	9 (4.7)	11 (5.8)	21 (3.7)
Pararesthesia	1 (0.5)	1 (0.5)	18 (9.5)	20 (3.5)
Dysgeusia	1 (0.5)	3 (1.6)	9 (4.7)	13 (2.3)
Headache	0	5 (2.6)	3 (1.6)	8 (1.4)
COVID-19	3 (1.6)	4 (2.1)	3 (1.6)	10 (1.8)
Urinary tract infection	3 (1.6)	2 (1.0)	4 (2.1)	9 (1.6)
Insomnia	1 (0.5)	10 (5.2)	8 (4.2)	19 (3.4)
Anxiety	1 (0.5)	5 (2.6)	5 (2.6)	11 (1.9)
Participants with TEAE leading to study drug discontinuation	4 (2.2)	11 (5.8)	18 (9.5)	33 (5.8)
Participants with TEAE leading to death	0	0	0	0

Abbreviations: MedDRA = Medical Dictionary for Regulatory Activities; TEAE = treatment-emergent adverse event.

The percentage is based on the number of participants under each treatment group in the Safety Population.

Multiple events are counted once per participant for each summary level.

Adverse events are coded using MedDRA version 24.1.

## 4 Discussion

This was a multicenter, randomized, double-blind ABPM study that evaluated the effects of PHEN/TPM on SBP compared to either placebo or phentermine. The per protocol primary analysis demonstrated a LS mean change from baseline to week 8 in SBP of −0.1 ​mmHg for the placebo group, +1.4 ​mmHg for the phentermine 30 ​mg group, and −3.3 ​mmHg for the PHEN/TPM group. The between-group difference for PHEN/TPM versus placebo was −3.2 ​mmHg (95 ​% CI: −5.48, −0.93 ​mmHg; p ​= ​0.0059), which met study-specified margins for both non-inferiority and superiority. The between-group difference for PHEN/TPM versus phentermine 30 ​mg was −4.7 ​mmHg (95 ​% CI: −6.96, −2.45 ​mmHg; p ​< ​0.0001), which also met the margins for both non-inferiority and superiority. The between-group difference for phentermine 30 ​mg versus placebo was 1.5 ​mmHg (95 ​% CI: −0.73, 3.73 ​mmHg; p ​= ​0.1867), which did not meet the margins for non-inferiority.

For secondary endpoints, while the comparison between PHEN/TPM and placebo for in-clinic SBP yielded results that were similar to the SBP response by 24-h ABPM, the results for mean 24-h ABPM DBP and in-clinic DBP met the threshold for non-inferiority but not the threshold for superiority. Accordingly, while comparisons for other endpoints based on ABPM assessments (daytime, nighttime, hourly, etc.) did show some differences between treatment groups, these comparisons beyond the primary and secondary endpoints should be viewed as exploratory.

The most commonly occurring TEAEs were in the system organ class of gastrointestinal disorders, nervous system disorders, infections and infestations, and psychiatric disorders, and were mild or moderate in severity. Overall, 5.8 % of participants had TEAEs causing discontinuation from study treatment (2.2 %, 5.8 %, and 9.5 % of participants in the placebo, phentermine 30 mg, and PHEN/TPM groups, respectively); the majority were considered related to study drug. The observed TEAEs were consistent with the known effects of PHEN/TPM and phentermine 30 mg, and do not suggest the presence of any new risks. Observed shifts in clinical chemistry parameters, specifically for carbon dioxide (normal to low) and creatinine (normal to high), were consistent with previous data on PHEN/TPM. Similarly, the increase in heart rate with PHEN/TPM as measured by 24-h ABPM were less than those observed with phentermine 30 mg (2.6 vs. 6.2 bpm) and consistent with previous assessments of in-clinic heart rate.

PHEN/TPM was associated with a significantly greater reduction of mean 24-h SBP than both placebo and phentermine 30 mg. A similar effect was observed for in-clinic SBP. For changes in DBP, PHEN/TPM was non-inferior to placebo, both for mean 24-h DBP by ABPM and in-clinic DBP. For this study participants were stratified according to hypertensive status (no hypertension, hypertension treated with 0–2 medications, and hypertension treated with 3 or more medications). Analyses of mean SBP by 24-hr ABPM were performed across these strata, and demonstrated consistent reduction of SBP across hypertensive strata, with no diminution of the blood pressure-reducing effect as the level of background medications increased. Taken together, these observations support PHEN/TPM as having beneficial effects on SBP compared to placebo as measured by ABPM in this population, as well as beneficial effects compared to phentermine 30 mg monotherapy.

Finally, while not the primary endpoint, this study evaluated higher doses of phentermine as a comparator. As previously noted, challenges exist in locating published reports of the blood pressure and heart rate effects of phentermine within the context of a prospective, blinded, randomized, controlled trial. In this study, compared to placebo at 8 weeks, phentermine 30 mg/day monotherapy increased systolic blood pressure by 1.5 mmHg (95 % CI: −0.73, 3.73 mmHg; p = 0.1867). Also at 8 weeks, participants in the phentermine 30 mg group had an increase in heart rate of 6.2 bpm compared to those in the placebo group who had a decrease in heart rate by 1 bpm.

### 4.1 Strengths and limitations

A strength of this study is that it provides the first assessment of the effects of PHEN/TPM on BP as measured by 24-h ABPM. While previous trials evaluating PHEN/TPM have reported statistically significant SBP reductions vs. placebo of 3–8 mmHg, these results all describe BP measured in-clinic [12], [13], [14], [15]. ABPM data on BP collected in this study provide a more comprehensive assessment of blood pressure control throughout the day and night, remove artifacts associated with white coat hypertension, and correlate more strongly with CV risk than spot measurements obtained in-clinic [16]. Moreover, control groups for this study include both placebo and phentermine 30 mg, with phentermine 30 mg being a widely used short term treatment for obesity despite little information regarding blinded, controlled clinical trial effects on blood pressure and heart rate [8]. Another strength is that the participant population for this study is representative of patients using PHEN/TPM commercially. Given the requirement for overweight and at least 1 weight-related complication, such patients would be expected to be at increased risk for cardiovascular disease. Finally, another strength of this analysis is the randomized, double-blinded, prospective study design. Prior reports of higher dose phentermine have predominantly been largely limited to retrospective health record reviews.

Limitations of this study include that although participants with controlled hypertension were included, those with baseline blood pressure greater than 140/90 were excluded. While it is encouraging that BP reductions in this study were numerically greater in participants with hypertension than those without, inclusion of more participants with elevated BP at baseline would have provided additional valuable information.

Also, a greater than anticipated number of participants were excluded from the per protocol population used for the primary analysis. This was due to participants’ reluctance or inability to properly complete end of study ABPM assessments. Had fewer participants been excluded, the power to evaluate secondary and exploratory endpoints may have been improved. Finally, this study did not evaluate longer-term effects of the comparator groups on blood pressure and heart rate, which may have changed with potential further weight reduction [17]. That said, an important objective of this study was to assess any potential for a pressor effect of study medications where the use of other concomitant antihypertensive medications remained constant, so that results were not confounded by additions or subtractions of these medications. While the study duration was limited to 8 weeks, the shorter study period better allowed an assessment of blood pressure while concomitant antihypertensive medications remained stable throughout the study period.

## 5 Conclusion

Compared to baseline, treatment with PHEN/TPM reduced mean SBP from baseline to week 8 as measured by 24-h ABPM compared to both placebo and to phentermine 30 mg monotherapy. Clinical takeaway messages:

• One of the more common adverse consequences of obesity is high blood pressure.
• High blood pressure is a cardiovascular disease risk factor.
• PHEN/TPM reduces the cardiovascular disease risk factor of SBP in patients with overweight or obesity.

## CRediT author statement

The concept and methodology of this submission was by CAP, LTN, and STV.

Statistical analysis and data curation was performed by CAP and LTN.

First draft writing was by LTN, CAP, and HEB.

HEB served as a Principal Investigator for this study.

All authors reviewed, edited subsequent versions, and approved the final submission.

## Source of funding

This study was funded by Vivus LLC.

## Ethics review

The study was initiated after approval of the Institutional Review Board, after proper consent from the participants, and conducted in compliance with the principles of the Declaration of Helsinki, International Conference on Harmonisation (ICH), Good Clinical Practices (GCP) guidelines, and applicable US Code of Federal Regulations. The peer review process took place via submission to the Executive Editor of Obesity Pillars, independent of any involvement of authors of this submission. ClinicalTrials.gov: NCT05215418.

## Declaration of artificial intelligence (AI) and AI-assisted technologies in the writing process

The authors did not use AI-assisted technologies during the preparation of this work.

## Declaration of competing interest

HEB's research site institution has received research grants from 89Bio, Alon Medtech/Epitomee, Altimmune, Amgen, Boehringer Ingelheim, Eli Lilly, Kallyope, Novartis, NovoNordisk, Pfizer, Shionogi, Viking, and Vivus. HEB has served as a consultant/advisor for 89Bio, Altimmune, Amgen, Boehringer Ingelheim, and Lilly.

DSH served as a site principal investigator for this study, which was conducted through a contract with his institution. DSH did not receive any funds directly from the sponsor. LTN is a full-time employee of VIVUS LLC, which markets the PHEN/TPM product evaluated in this study. At the time of this study her work on this study was done as part of her employment.

CAP is a full-time employee of VIVUS LLC, which markets the PHEN/TPM product evaluated in this study. At the time of this study, his work on this study was done as part of his employment.

STV is a full-time employee of VIVUS LLC, which markets the PHEN/TPM product evaluated in this study. At the time of this study, his work on this study was done as part of his employment and role as an officer in the company.

## References

[1] Clayton T.L., Fitch A., Bays H.E. (2023). Obesity and hypertension: obesity medicine association (OMA) clinical practice statement (CPS) 2023. Obesity Pillars.

[2] Hall J.E., Carmo JMd, Silva AAd, Wang Z., Hall M.E. (2015). Obesity-induced hypertension. Circ Res.

[3] Hall M.E., Cohen J.B., Ard J.D., Egan B.M., Hall J.E., Lavie C.J. (2021). Weight-loss strategies for prevention and treatment of hypertension: a scientific statement from the American heart association. Hypertension.

[4] Bays H. (2010). Phentermine, topiramate and their combination for the treatment of adiposopathy (’sick fat’) and metabolic disease. Expet Rev Cardiovasc Ther.

[5] Tonstad S., Tykarski A., Weissgarten J., Ivleva A., Levy B., Kumar A. (2005). Efficacy and safety of topiramate in the treatment of obese subjects with essential hypertension. Am J Cardiol.

[6] Pearl N.Z., Babin C.P., Catalano N.T., Blake J.C., Ahmadzadeh S., Shekoohi S. (2023). Narrative review of topiramate: clinical uses and pharmacological considerations. Adv Ther.

[7] Leaf D.E., Goldfarb D.S. (1985). Mechanisms of action of acetazolamide in the prophylaxis and treatment of acute mountain sickness. J Appl Physiol.

[8] Bays H.E., Lazarus E., Primack C., Fitch A. (2022). Obesity pillars roundtable: phentermine - Past, present, and future. Obes Pillars.

[9] Bays H.E., Kulkarni A., German C., Satish P., Iluyomade A., Dudum R. (2022). Ten things to know about ten cardiovascular disease risk factors - 2022. Am J Prev Cardiol.

[10] Aronne L.J., Wadden T.A., Peterson C., Winslow D., Odeh S., Gadde K.M. (2013). Evaluation of phentermine and topiramate versus phentermine/topiramate extended-release in obese adults. Obesity.

[11] Bays H.E., Burridge K., Richards J., Fitch A. (2022). Obesity Pillars roundtable: excessive weight reduction with highly effective anti-obesity medications (heAOMs). Obes Pillars.

[12] Allison DB, Gadde KM, Garvey WT (2012). Controlled-release phentermine/topiramate in severely obese adults: a randomized controlled trial (EQUIP). Obesity (Silver Spring).

[13] Gadde KM, Allison DB, Ryan DH (2011). Effects of low-dose, controlled-release, phentermine plus topiramate combination on weight and associated comorbidities in overweight and obese adults (CONQUER): a randomised, placebo-controlled, phase 3 trial. Lancet.

[14] Garvey WT, Ryan DH, Bohannon NJ (2014). Weight-loss therapy in type 2 diabetes: effects of phentermine and topiramate extended release. Diabetes Care.

[15] Winslow DH, Bowden CH, DiDonato KP, McCullough PA (2012). A randomized, double-blind, placebo-controlled study of an oral, extended-release formulation of phentermine/topiramate for the treatment of obstructive sleep apnea in obese adults. Sleep.

[16] Verdecchia P, Angeli F, Cavallini C (2007). Ambulatory blood pressure for cardiovascular risk stratification. Circulation.

[17] Lewis KH, Fischer H, Ard J, Barton L, Bessesen DH, Daley MF (2019). Safety and Effectiveness of Longer-Term Phentermine Use: Clinical Outcomes from an Electronic Health Record Cohort. Obesity (Silver Spring, Md).

