# Supplementary material for: Effects of phentermine / topiramate extended-release, phentermine, and placebo on ambulatory blood pressure monitoring in adults with overweight or obesity: A randomized, multicenter, double-blind study
Source: Obes Pillars. 2024 Jan 8;9:100099. doi: 10.1016/j.obpill.2024.100099 (PMC10831272; doi:10.1016/j.obpill.2024.100099)
Supplement: Multimedia component 1 [file mmc1.docx]

**SUPPLEMENTARY APPENDIX**

**Inclusion/Exclusion Criteria**

**Inclusion Criteria**

To be eligible for enrollment into this study, participants must meet all of the following criteria at Screening (unless otherwise specified):

1. Overweight or obese adult males and females 18-75 years of age with a BMI ≥ 27 kg/m^2^;
2. Medical diagnosis of at least 1 weight-related comorbidity (i.e., hypertension, dyslipidemia, type 2 diabetes mellitus or prediabetes, or obstructive sleep apnea);
3. Must be ambulatory, willing, and able to wear ABPM monitor apparatus for 24 hours during two study measuring visits, at Baseline and Week 8/end of study or early termination;
4. Screening laboratory values that are within the ranges specified below:

| 1. Bicarbonate | ≥ LLN |
| --- | --- |
| 1. AST and ALT | < 3 x ULN |
| 1. HbA1c | ≤ 7.5% |
| 1. TSH | ≤ 1.5 x ULN |
| 1. Triglyceride | ≤ 400 mg/dL |
| 1. Creatinine clearance | ≥ 60 mL/minute (MDRD) |

1. Females of childbearing potential must be using adequate contraception, defined as double barrier methods, stable hormonal contraception plus single barrier method, or previously documented bilateral tubal ligation. Women are considered of childbearing potential unless they are ≥ 50 years of age with spontaneous amenorrhea for at least 12 months or have had a hysterectomy and/or bilateral oophorectomy;
2. Provide written informed consent; and
3. Willing and able to comply with scheduled study visits, treatment plan, laboratory tests, and other study procedures.

**Exclusion Criteria**

To be eligible for enrollment into this study, participants must not meet any of the following criteria at screening (unless otherwise specified):

1. Screening blood pressure of > 140/90 mmHg;
2. Known allergy or hypersensitivity to phentermine or topiramate, any prior use of a combination of phentermine and topiramate for weight loss or use of phentermine or topiramate for any indication within the past 3 months;
3. Weight gain or loss of greater than 5 kg, use of a very low-calorie diet, or participation in a formal weight loss program (investigational or otherwise) within the past 3 months (this includes: Weight Watchers and related dietary/lifestyle intervention programs; prepared food programs; prescribed or over-the-counter weight loss medications; dietary supplement or herbal preparations, teas, or tinctures intended for weight loss; or any medically-supervised fast or very low calorie diet);
4. Obesity of a known genetic or endocrine origin;
5. Previous bariatric surgery or other non-surgical weight loss procedure;
6. History of any eating disorders (e.g., bulimia, binge eating disorder) within the past year;
7. History or presence of a seizure disorder;
8. History of drug or alcohol abuse within the past year or positive drug test;
9. Smoking cessation within the past 3 months or intent to quit during the study;
10. Use of antihypertensive medications, antidiabetic medications, statins or other lipid lowering agents, or CPAP therapy that has not been stable for at least 3 months prior to randomization;
11. Chronic conditions/diseases associated with a reduced ability to maintain autonomic regulation such as multiple sclerosis or any known autonomic neuropathy;
12. History of glaucoma, increased intraocular pressure, or any past or present use of medications to treat increased intraocular pressure;
13. Clinical evidence of hyperthyroidism, or use of thyroid hormone treatment that has not been stable for at least 3 months prior to randomization;
14. Use of chronic systemic glucocorticoid therapy, or any other steroid hormone therapy that has not been stable for at least 3 months at the time of randomization;
15. Any history of bipolar disorder or psychosis, greater than one lifetime episode of major depressive disorder, or presence or any history of suicidal behavior or suicidal ideation with some intent to act; any use of tricyclic antidepressants, monoamine oxidase inhibitors (MAOIs), lithium, levodopa, or dopamine receptor agonists; or allowed antidepressant use that has not been stable for at least 3 months;
16. Need to perform strenuous manual labor or exercise while wearing the ABPM monitor during the 24-hr periods when those measurements are being recorded;
17. Night shift workers who routinely sleep during the daytime and whose work hours include midnight to 4:00 am, or workers who are subject to assignment to different shifts during the study;
18. Diagnosis of type 1 diabetes; type 2 diabetes mellitus with ongoing insulin, sulfonylureas (SFUs), GLP-1 receptor agonist, or SGLT inhibitor therapy;
19. Stroke, myocardial infarction, or coronary revascularization within the past 6 months;
20. Presence of cardiac pacemaker or implantable defibrillator;
21. Presence or history of clinically significant atrial fibrillation or atrial flutter, AV block > 1st degree, ventricular ectopy, or sustained ventricular tachycardia;
22. Unstable angina, congestive heart failure (NYHA Class III or IV), or known or suspected cardiac valvulopathy;
23. Presence of asthma or COPD (chronic obstructive pulmonary disease) requiring routine or rescue treatment with an adrenergic bronchodilator;
24. Any history of malignancy within the past 5 years other than surgically excised basal or squamous cell carcinoma of the skin or cervical cancer;
25. Cholelithiasis within the past 6 months;
26. Any history of nephrolithiasis;
27. Use of any investigational medication or device for any indication within a month prior to randomization;
28. COVID-19 vaccination or treatment for severe COVID-19 infection within a month prior to randomization; or
29. Clinically significant renal, pulmonary, hepatic, psychiatric or other condition by history, physical examination or laboratory studies that, in the opinion of the investigator, would contraindicate the administration of study drugs, affect compliance, interfere with study evaluations or confound the interpretation of study results.
